# Robot-Assisted Gait Training Enhances Phase-Specific Torque Generation, Balance, and Motor Recovery in Hemiplegia

**DOI:** 10.3390/s26102920

**Published:** 2026-05-07

**Authors:** Gökhan Özkoçak, Ecem Sorucu, Rocco Salvatore Calabrò

**Affiliations:** 1 Department of Physical Medicine and Rehabilitation, Faculty of Medicine, Istanbul Aydin University, 34295 Istanbul, Turkey; 2 Faculty of Life Sciences, University of Bristol, Bristol BS8 1QU, UK; 3 Spinal Cord Unit, IRCCS (Istituto di Ricovero e Cura a Carattere Scientifico) Centro Neurolesi “Bonino-Pulejo”, 98124 Messina, Italy

**Keywords:** stroke, hemiplegia, robot-assisted gait training, gait biomechanics, swing phase, stance phase, joint torque, Berg Balance Scale, Fugl–Meyer Assessment, motor recovery

## Abstract

Gait dysfunction is a common and disabling consequence of stroke, frequently associated with impaired lower-limb torque generation and reduced balance. Robot-assisted gait training (RAGT) has emerged as a promising intervention; however, its phase-specific biomechanical effects remain incompletely characterized. This pilot mechanistic study investigated the effects of Walkbot-assisted gait training on phase-specific lower-limb torque and clinical outcomes in individuals with unilateral hemiplegia. Fifteen patients with hemiplegia underwent Walkbot-assisted gait training. Joint torque values were normalized to body mass (Nm/kg). Phase-specific torque was analyzed during the swing and stance phases for the affected and unaffected limbs. Pre–post differences were evaluated using the Wilcoxon signed-rank test. Functional balance and motor impairment were assessed using the Berg Balance Scale (BBS) and the Fugl–Meyer Assessment—Lower Extremity (FMA-LE). Significant torque increases were observed in both gait phases. Median swing-phase torque increased from 0.261 to 0.361 Nm/kg in the affected limb and from 0.254 to 0.334 Nm/kg in the unaffected limb (*p* ≤ 0.017). Stance-phase torque increased from 0.197 to 0.454 Nm/kg in the affected limb and from 0.158 to 0.471 Nm/kg in the unaffected limb. Clinical outcomes improved significantly, with median BBS scores increasing from 22.0 to 34.0 and FMA-LE scores from 14.0 to 24.0 (*p* = 0.001). Walkbot-assisted gait training was associated with significant phase-specific torque gains, accompanied by improvements in balance and lower-limb motor recovery.

## 1. Introduction

Gait impairments are among the most persistent and functionally limiting consequences of stroke, affecting more than 60% of survivors and contributing significantly to reduced independence, increased fall risk, and long-term disability [1,2]. Advances in rehabilitation technologies have led to the emergence of robot-assisted gait training (RAGT), with systems like Walkbot offering promising solutions to enhance motor recovery through task-specific, repetitive, and feedback-enriched gait therapy. The Walkbot exoskeletal system enables lower-limb joint assistance during overground walking, with adjustable parameters such as body-weight support, gait speed, and impedance, potentially allowing for tailored neuromuscular engagement and therapeutic progression.

Since its inception in the early 2000s, RAGT has transitioned from passive treadmill-based models to sophisticated, sensor-integrated robotic exoskeletons that promote dynamic joint control and neuroplastic adaptation [3,4]. These robotic interventions have been shown to influence not only gross motor outcomes but also biomechanical parameters, such as joint torque, moment generation, and interlimb coordination, which are crucial for restoring physiological gait patterns [5,6]. However, despite an expanding clinical literature, mechanistic evidence remains comparatively limited, as many studies prioritize spatiotemporal or kinematic outcomes and report fewer kinetic endpoints (e.g., phase-specific joint torques) that directly reflect force-generating capacity during gait [5,6]. This limits cross-study comparability and constrains understanding of which gait subcomponents (e.g., limb advancement vs. weight acceptance) are most responsive to robotic training.

Among kinetic outcomes, knee-joint moments—particularly active extension/flexion torque during stance (limb bearing weight) and swing (limb in the air)—have received growing attention due to their direct relevance to limb advancement, load-bearing, and push-off mechanics. The stance and swing phases represent biomechanically distinct functional demands and therefore provide a useful framework for interpreting phase-dependent joint moment adaptations. The stance phase is the period when the foot contacts the ground and body weight is carried; weight transfer, stability, and propulsion are generated during this phase [7]. The swing phase is the period when the foot leaves the ground and advances forward; coordinated knee flexion and ankle dorsiflexion contribute to step normalization and foot clearance [8].

During the stance phase, the paretic limb typically exhibits a marked decrease in knee extensor torque, impairing safe weight transfer, together with reduced ankle plantar-flexor torque, resulting in insufficient push-off during terminal stance. Hip extensor torque may also be reduced and/or poorly timed in many patients. In contrast, the non-paretic limb often demonstrates compensatory increases in knee and hip extensor torque throughout stance. While such compensation may support short-term stability, it may also increase mechanical loading on the non-paretic limb and contribute to persistent asymmetry over time.

Walkbot-assisted training provides repeated, phase-specific stepping practice that may facilitate neuromuscular re-education; however, the biomechanical mechanisms underlying its effects remain incompletely understood. In particular, whether Walkbot training can modify phase-specific torque generation patterns and reduce kinetic asymmetry in individuals with post-stroke hemiplegia remains unclear. Existing research suggests that the timing of intervention, stroke chronicity, and individual response variability further complicate the interpretation of outcomes and optimal therapy dosing [2,9]. The lack of standardized biomechanical outcome measures, together with limited long-term follow-up, constrains the ability to synthesize data and draw robust conclusions across studies.

In light of these gaps, the present study aims to evaluate the impact of Walkbot-assisted gait training on swing- and stance-phase performance by quantifying phase-specific lower-limb torque generation in the affected and unaffected limbs during these phases, and by determining whether torque changes are accompanied by improvements in functional balance, as assessed using the Berg Balance Scale (BBS).

Beyond biomechanical deficits, stroke-related gait dysfunction is strongly influenced by lower-limb motor impairment. The Fugl–Meyer Assessment—Lower Extremity (FMA-LE) is widely used to quantify stroke-specific motor recovery, evaluating reflex activity, movement synergies, and selective voluntary control. Because improvements in joint torque may reflect either compensatory strategies or true motor restitution, incorporating an impairment-level measure such as the FMA-LE provides critical clinical context for interpreting biomechanical adaptations.

By leveraging the sensor-integrated architecture of the Walkbot system, the present study utilizes joint-level kinetic and kinematic signals as objective biomarkers of gait adaptation, reflecting the growing role of embedded sensing technologies in quantitative neurorehabilitation research.

To the best of our knowledge, this study is the first to evaluate phase-specific torque characteristics during robot-assisted gait training in stroke rehabilitation in conjunction with established clinical outcome measures. By integrating gait-phase-dependent neuromechanical outputs with clinical assessment parameters, this study moves beyond conventional functional reporting and provides a more comprehensive understanding of how robotic gait training influences both observable recovery and its underlying biomechanical mechanisms.

## 2. Materials and Methods

This was a retrospective, single-group, pre–post interventional study evaluating the biomechanical effects of Walkbot-assisted gait training in patients with hemiplegic stroke. It followed STROBE guidelines and was approved by the University Ethics Committee (Approval No. E-10840098-202.3.02-5668). Written informed consent for participation in the study was obtained from all patients. All stages of the study were conducted in accordance with the ethical principles of the Declaration of Helsinki.

**Figure 1:**
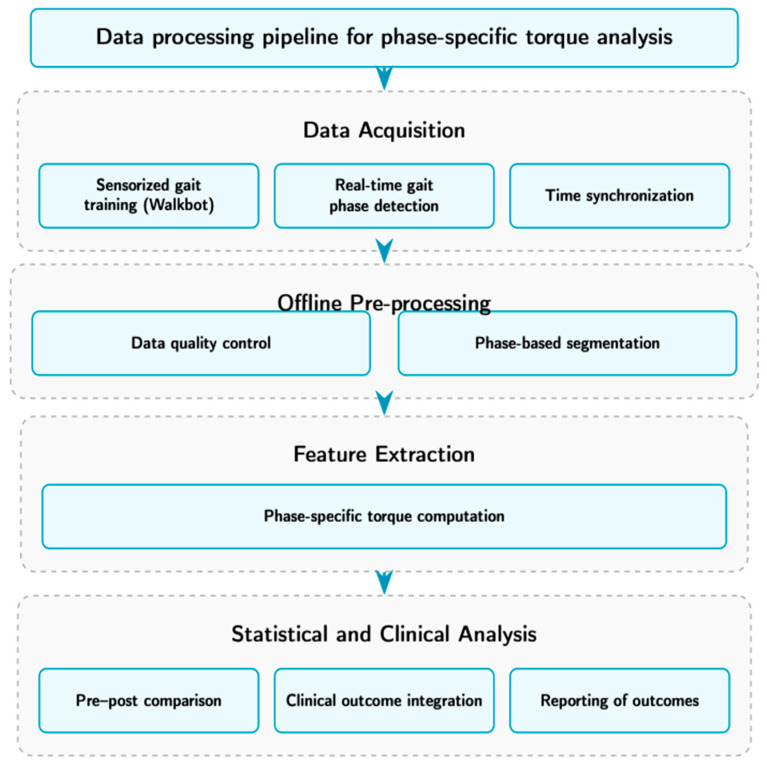
Data-processing pipeline for phase-specific torque analysis.

**Figure 2:**
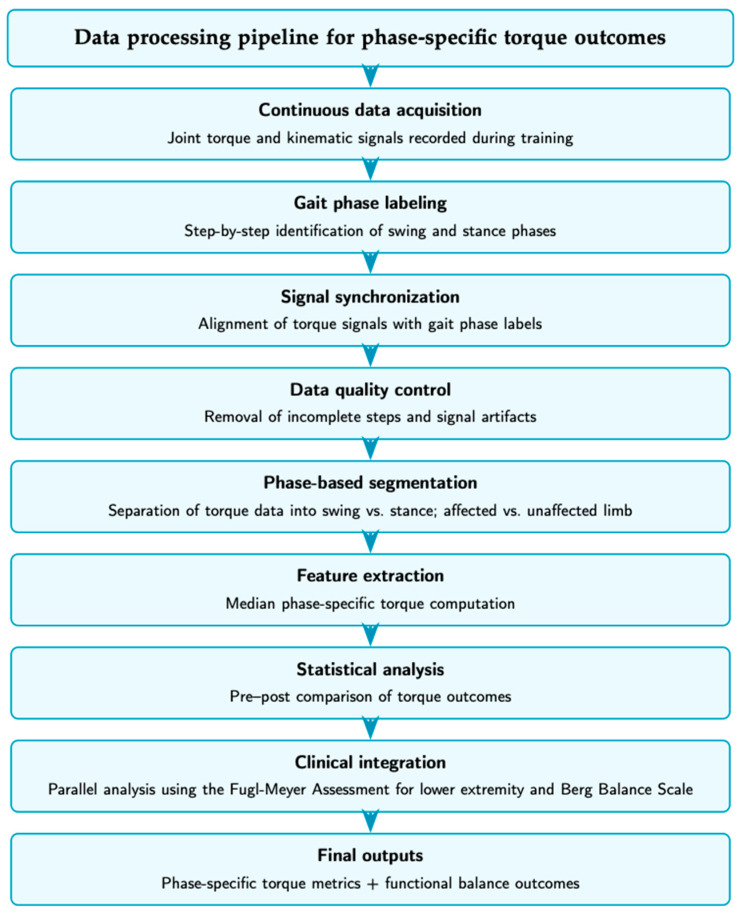
Data-processing pipeline for phase-specific torque outcomes.

The overall workflow of this study, including data acquisition, signal processing, feature extraction, and statistical evaluation, is summarized in the flowchart. As illustrated, joint torque and kinematic signals were first acquired during Walkbot-assisted gait training using the integrated sensor system. Gait phases were identified in real time and synchronized with torque signals, followed by offline data quality screening and phase-based segmentation. Phase-specific torque values were then computed for the affected and unaffected limbs during the swing and stance phases. Finally, the extracted biomechanical measures were analyzed using non-parametric pre–post comparisons and interpreted together with clinical outcome measures. This structured pipeline was designed to ensure transparency, reproducibility, and a clear linkage between sensor-derived biomechanical data and functional outcomes (Figure 1 and Figure 2).

### 2.1. Participants

This study was conducted with a total of *n* = 15 adult participants diagnosed with hemiplegia due to stroke.

The participants were recruited between July 2024 and June 2025. The inclusion criteria were as follows:

- Age: 40–75 years;
- Clinical diagnosis of hemiplegia < 6 months post-stroke;
- Ability to follow verbal instructions (Mini-Mental State Examination score≥24).

The exclusion criteria were as follows:

- Fixed joint contractures or orthopedic impairments affecting gait;
- Severe cardiopulmonary instability;
- History of recent lower-limb surgery (<6 months);
- Severe cognitive or behavioral impairments.

Participants completed a 5-week Walkbot training protocol using the Walkbot-G robotic gait system (P&S Mechanics, Seoul, Republic of Korea). Each session lasted an average of 45 min or until the patient took 1000 steps and was conducted 3 times per week (15 sessions total). Training parameters—body-weight support, step length, and walking speed—were individualized and adjusted throughout the program by an experienced physiotherapist. No concurrent therapy was administered during the study period.

### 2.2. Biomechanical Measurements

**Figure 3:**
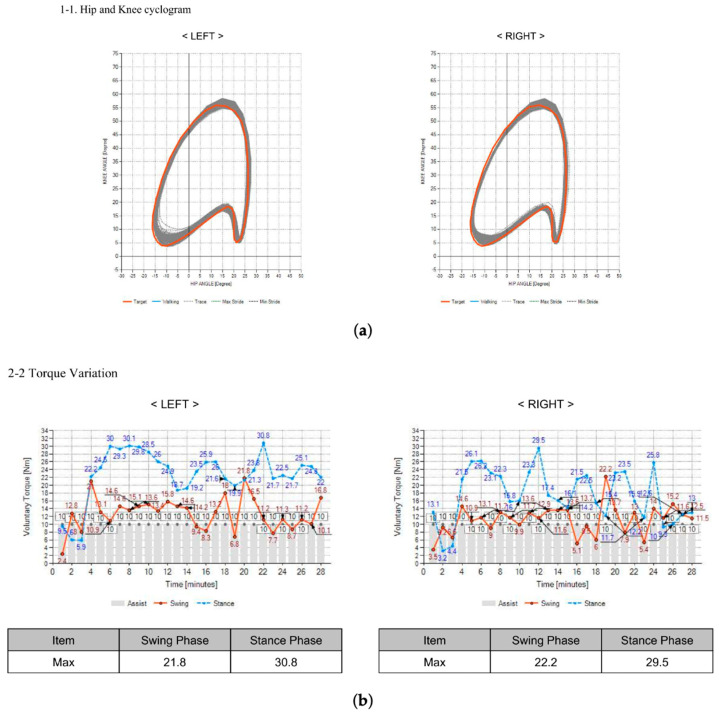
(**a**) Hip and knee cyclogram. (**b**) Example of voluntary torque changes in the left and right swing and stance phases.

Bilateral knee-joint torque values were recorded using the Walkbot’s integrated sensor system, which captures joint kinetics throughout the gait cycle. Data were extracted for two gait phases: swing and stance (Figure 3).

Measurements were taken during steady walking at two time points: pre-intervention (first session) and post-intervention (last session). Data were classified into affected and unaffected legs based on the side of hemiplegia. Total torque values were calculated for each patient.

During Walkbot-assisted sessions, sensor-derived torque streams were continuously logged and synchronized with real-time gait-phase labels (swing/stance). Offline processing included data quality screening and phase-based segmentation to isolate swing- and stance-specific torque samples for the affected and unaffected limbs. For each participant, phase-specific torque outcomes were summarized using medians and dispersion measures, and pre–post differences were evaluated using non-parametric paired testing.

Biomechanical data processing was based on the integrated Walkbot system outputs. Joint torque signals were recorded continuously, and gait phases were identified in real time by the device’s embedded sensor-based processing system. Because the detailed internal phase-detection algorithm is proprietary, the exact threshold values and computational decision rules were not available for independent reporting. Accordingly, the present analysis used the exported swing/stance labels generated by the system.

After export, the data underwent offline review for completeness and signal quality. Segments corresponding to incomplete gait cycles or obvious recording artifacts were excluded from the summary analysis. The remaining data were grouped by gait phase and limb side, and median torque values were calculated separately for swing and stance in the affected and unaffected limbs.

Functional balance was evaluated using the BBS, a validated and widely used measure of static and dynamic balance. The BBS consists of 14 performance-based tasks assessing postural control and balance during functional activities. Scores range from 0 to 56, with higher scores indicating better balance performance.

Motor impairment of the paretic lower limb was evaluated using the FMA-LE, a validated and widely used stroke-specific measure of sensorimotor recovery. The FMA-LE assesses reflex activity, movement synergies, and selective voluntary motor control, with total scores ranging from 0 to 34. Higher scores represent less motor impairment.

### 2.3. Statistical Analysis

All statistical analyses were conducted using SPSS (version 26; IBM Corp., Armonk, NY, USA). Given the small sample size and non-normal distribution of variables, continuous data are reported as medians (IQR). Within-subject pre–post differences were evaluated using the Wilcoxon signed-rank test. Statistical significance was defined as a two-tailed *p*-value < 0.05.

An overview of the sensor-based architecture and measurement capabilities of the Walkbot system is provided in Table 1, highlighting its role as both a rehabilitation device and a quantitative gait assessment platform.

### 2.4. Walkbot System Architecture and Sensor-Based Capabilities

The Walkbot robotic gait training system is a sensor-integrated lower-limb exoskeleton developed to deliver programmable, phase-specific assistance during gait while simultaneously enabling objective biomechanical data acquisition. Beyond its therapeutic role, Walkbot functions as a sensor-based measurement platform, aligning with the increasing emphasis on wearable and embedded sensors for quantitative assessment in neurorehabilitation research [10,11].

Walkbot is a robot-assisted gait training system designed for body-weight-supported treadmill rehabilitation using a lower-limb exoskeletal structure. Across available technical and clinical descriptions, Walkbot is presented as a system that combines partial body-weight support, sagittal-plane robotic gait guidance, and real-time biomechanical feedback during locomotor training. In published studies, the Walkbot_G version is described as having six actuated orthoses attached to the patient’s lower limbs with cuffs and straps, with the hip, knee, and ankle joints driven through the gait cycle in the sagittal plane.

A central feature of Walkbot is that it is not only a training device but also a measurement-enabled rehabilitation platform. One study reports that hip and knee torques can be determined using force sensors located between the actuators and the orthoses, which is one of the clearest published statements about Walkbot’s internal sensing architecture. The same study states that the front monitor provides real-time visual feedback on active and resistive torques and kinematics for the hip and knee joints during training [6].

From a biomechanical perspective, this means that Walkbot integrates at least three functional layers. First, it provides robotic joint guidance at the lower extremities. Second, it provides body-weight unloading through an external harness-based support system. Third, it provides quantitative joint-level biofeedback that can be used to monitor gait-related kinetics and kinematics during training. The literature further supports this interpretation by describing Walkbot as a system equipped with a 6-degree-of-freedom actuator-controlled exoskeleton for the hip, knee, and ankle joints, augmented by virtual reality and adjustable partial body-weight suspension support. That paper also states that the system offers real-time visual and haptic biofeedback for torque, kinematics, and active and resistive stiffness of hip, knee, and ankle joint movements [12].

Based on these studies, the most directly documented sensor-related features are located at the hip and knee modules. The Walkbot_G study explicitly states that force sensors between the actuators and orthoses are used to derive knee and hip torques. This is important because it shows that Walkbot does not rely solely on trajectory replay; it also captures interaction-related kinetic information during stepping.

The aforementioned study adds another layer of detail by explaining that Walkbot uses servomotors and specialized encoders to control the trajectories of the hip, knee, and ankle joints. These encoders record joint angle, angular velocity, and acceleration signals, which are then filtered and transformed to calculate moment-related variables associated with active and resistive forces acting on the body segments during walking. Although the paper emphasizes hip and knee stiffness/moment outcomes, it clearly indicates that the underlying kinematic sensing infrastructure spans the three lower-limb joints.

At the ankle level, the study reports that spring-loaded cloth straps are added to induce ankle dorsiflexion during the swing phase. This is a directly reported mechanical feature. However, compared with hip and knee sensing, the paper does not explicitly document an ankle force sensor in the same way. Therefore, the safest interpretation is that the ankle module is clearly active in gait assistance and kinematic coordination, but the public evidence for dedicated ankle torque sensing is less explicit than it is for the hip and knee.

The study explicitly states that Walkbot uses an impedance-based control mode. In this framework, the device acts as an interactive resistive–assistive system, allowing the patient to move either with or against the impedance-based forces generated by the exoskeleton. The authors explain that at lower impedance levels, greater resistive-assistive interaction is provided while the patient actively attempts to achieve target movement, whereas higher impedance is used to guide the lower limbs along a more predefined trajectory. The same section states that Walkbot provides concurrent real-time information related to ankle–knee–hip segmental kinematic and kinetic coordination [6].

This makes Walkbot biomechanically relevant not only as a repetitive gait machine but also as a patient-cooperative training system. In practice, this means that the device can be interpreted as operating somewhere between fully imposed robotic stepping and partially interactive motor training, depending on how impedance and assistance are set.

The study provides a clinically useful description of how Walkbot is configured for each patient. It states that anthropometric and clinical data are encoded into the Walkbot system to create optimal gait kinematic and kinetic parameters for the individual. The patient then enters the device, dons the suspension harness, and the hip, knee, and ankle joints are aligned with the corresponding exoskeletal actuators. Initial body-weight bearing is set at approximately 40–50%, with progression over sessions, and walking velocity begins at around 1.0–1.2 km/h and can be increased up to the patient’s tolerance, with a reported maximum of 3.0 km/h.

This is biomechanically important because correct alignment between anatomical joints and robotic axes is essential for reducing abnormal shear forces, improving torque transfer, and promoting more physiological segmental motion during assisted gait training. The fact that the system uses encoded anthropometric data supports the interpretation that Walkbot is designed for individualized lower-limb kinematic fitting, rather than a one-size-fits-all stepping pattern.

Walkbot’s sensing and control architecture appears particularly suited to monitoring and shaping several key gait-cycle events.

During stance-related phases, joint torques at the hip and knee, together with body-weight support settings, influence how much load the patient accepts and how the lower limb resists or assists the imposed trajectory. Because the system reports active and resistive torques, it can distinguish between movement generated in the same direction as the intended joint action and movement-resisting interaction in the opposite direction.

During swing, the ankle component becomes especially important. The study specifically notes the use of spring-loaded straps to promote ankle dorsiflexion during swing, which supports toe clearance and reduces the likelihood of drag. At the same time, hip and knee actuation continue to guide segmental progression through the gait cycle.

The results also demonstrate that Walkbot can detect clinically meaningful changes in gait-related biomechanics. After training, significant gains were reported in maximal hip flexion, maximal hip extension, and maximal knee flexion, together with increased active knee torque and reduced resistive hip torque. These findings suggest that Walkbot-based training can influence both kinematics and kinetics, not only observational gait quality.

### 2.5. Joint Actuation and Torque Sensing

Walkbot is equipped with bilaterally actuated hip, knee, and ankle joints, each driven by electric motors coupled with high-resolution joint encoders. Joint torque is estimated in real time using embedded torque sensors and motor current-based estimation algorithms, which have been shown to provide reliable proxies for joint-level kinetic output during robot-assisted walking.

The fundamental principle of motor current-based torque estimation, a standard approach in active robotic orthoses [6], can be expressed by the following governing equation:

(1) $$\tau_{est}=\left(K_{t}\cdot I\right)-\tau_{fric}-\tau_{grav}$$

where $\tau_{est}$ is the estimated joint torque, $K_{t}$ is the motor torque constant, I is the measured electric current, $\tau_{fric}$ represents internal system friction and $\tau_{grav}$ accounts for the gravitational compensation of the exoskeleton limbs.

To ensure cross-subject comparability according to standard biomechanical practices [7,13], the absolute joint torque values were normalized to each participant’s body mass. The normalized phase-specific torque ($\tau_{norm}$) was calculated as follows:

(2) $$\tau_{norm}=\frac{\tau_{est}}{m}$$

where m represents the participant’s body mass in kilograms (kg), yielding the final outcome unit of Nm/kg.

These torque signals are continuously sampled and synchronized with gait-phase information, allowing extraction of phase-specific torque profiles during swing and stance. Such joint-level kinetic data offer objective insight into force-generating capacity and neuromuscular engagement that cannot be captured by spatiotemporal measures alone [14]. Because absolute torque values are influenced by body weight, all joint torque measurements were normalized to body mass and reported as Nm/kg. This normalization improves comparability across individuals and enhances the physiological interpretability of kinetic outcomes.

### 2.6. Gait-Phase Detection via Sensor Fusion

Accurate segmentation of the gait cycle is achieved through multi-sensor fusion, combining joint angle trajectories, angular velocity signals, and foot–ground interaction cues derived from kinematic thresholds. This approach enables robust identification of stance and swing phases on a step-by-step basis and supports real-time modulation of robotic assistance [15].

Although exact internal threshold values are commercially confidential, the generalized sensor fusion logic used for real-time phase transitions can be mathematically conceptualized as a conditional state function, consistent with finite-state machine algorithms commonly described in exoskeleton research [16,17]:

(3) $$\mathrm{Phase}\left(t\right)=\left\{\begin{array}{ll} \mathrm{Stance}, & \mathrm{if}\;\theta_{kin}\left(t\right)\in S_{\mathrm{stance}}\;\mathrm{and}\;\omega \left(t\right)\leq \omega_{th}\\ \mathrm{Swing}, & \mathrm{if}\;\theta_{kin}\left(t\right)\in S_{\mathrm{swing}}\;\mathrm{and}\;\omega \left(t\right)>\omega_{th} \end{array}\right.$$

Here, $\theta_{kin}\left(t\right)$ represents the continuous multi-joint kinematic profile at time t; $S_{\mathrm{stance}}$ and $S_{\mathrm{swing}}$ are predefined reference kinematic spaces for each phase; and $\omega \left(t\right)$ is the angular velocity compared to temporal phase transition thresholds ($\omega_{th}$). This continuous Boolean evaluation ensures that joint torque data is accurately labeled and separated into the respective swing and stance phases.

Phase-resolved sensor data are stored and subsequently used for offline biomechanical analyses, facilitating comparison of torque and kinematic patterns across training sessions (Table 1).

### 2.7. Kinematic Sensing and Spatiotemporal Data Acquisition

High-resolution rotary encoders embedded at each joint provide continuous measurement of joint angles and angular velocities. From these signals, Walkbot computes kinematic variables and spatiotemporal gait parameters, including step timing, cadence, and joint range of motion. The synchronized acquisition of kinematic and kinetic data enables comprehensive characterization of gait mechanics and supports sensor-based evaluation of rehabilitation-induced adaptations (Table 1) [18,19].

### 2.8. Control Strategy and Programmable Assistance Parameters

Walkbot employs a hybrid position- and impedance-based control strategy, allowing adjustment of robotic assistance according to patient capability and therapeutic goals. Programmable parameters include body-weight support, gait speed, joint impedance, and assistance magnitude.

The underlying interaction between the patient and the Walkbot system during impedance control can be modeled using a standard proportional–derivative (PD) control law, a foundational framework in interactive rehabilitation robotics [20,21], determining the assistive torque provided by the robot:

(4) $$\tau_{robot}=K_{p}\left(\theta_{d}-\theta_{a}\right)+K_{d}\left({\dot{\theta }}_{d}-{\dot{\theta }}_{a}\right)$$

where $\tau_{robot}$ is the assistive torque generated by the actuator; $\theta_{d}$ and $\theta_{a}$ are the reference and actual joint angular positions, respectively. Similarly, ${\dot{\theta }}_{d}$ and ${\dot{\theta }}_{a}$ represent the desired and actual joint angular velocities. The parameters $K_{p}$ (virtual stiffness) and $K_{d}$ (virtual damping) can be adjusted to modulate assistance. By varying these parameters, the system can provide high assistance with stiff movement or low assistance with flexible, patient-controlled movement.

These parameters are logged by the system and can be linked to sensor-derived biomechanical outputs, enabling analysis of dose–response relationships between robotic assistance and gait adaptation. Gradual reduction of assistance promotes active patient participation while maintaining the safety and consistency of sensor measurements (Table 1) [10].

### 2.9. Data Logging and Integration with Clinical Outcomes

All sensor-derived signals—including joint angles, estimated torques, and gait-phase labels—are recorded continuously during training sessions. This facilitates post hoc computation of summary measures such as median torque, variability, and asymmetry across gait phases. Integration of sensor-based biomechanical measures with clinical assessments (e.g., balance and mobility scales) enables investigation of relationships between objective sensor outputs and functional recovery, supporting a data-driven approach to neurorehabilitation (Table 1) [22,23].

## 3. Results

Fifteen individuals with unilateral hemiplegia secondary to stroke participated in this study. The mean age of participants was 58 ± 7 years. The cohort consisted of nine males (60%) and six females (40%). In terms of stroke subtype, 33.3% of participants had ischemic stroke and 66.7% had hemorrhagic stroke (Table 2).

All continuous variables are reported as medians (minimum–maximum, [interquartile range]). Pre–post comparisons were performed using the Wilcoxon signed-rank test.

### 3.1. Swing Phase

Following Walkbot-assisted gait training, significant increases in swing-phase torque were observed in both the affected and unaffected limbs (Table 3). In the affected limb, median swing-phase torque increased from 0.261 Nm/kg (0.084–0.458, IQR = 0.161) at baseline to 0.361 Nm/kg (0.117–0.767, IQR = 0.216) post-training, corresponding to a median change of +0.095 Nm/kg (*p* = 0.017). Similarly, in the unaffected limb, median swing-phase torque increased from 0.254 Nm/kg (0.058–0.440, IQR = 0.131) to 0.334 Nm/kg (0.202–0.740, IQR = 0.182), yielding a median improvement of +0.136 Nm/kg (*p* = 0.011). These findings indicate that Walkbot-assisted training was associated with enhanced torque generation during the swing phase in both limbs.

### 3.2. Stance Phase

Significant torque increases were also observed during the stance phase following Walkbot-assisted gait training (Table 3). In the affected limb, median stance-phase torque increased from 0.197 Nm/kg (0.005–0.512, IQR = 0.232) at baseline to 0.454 Nm/kg (0.208–0.851, IQR = 0.350) post-training, corresponding to a median change of +0.197 Nm/kg (*p* = 0.001). Similarly, in the unaffected limb, median stance-phase torque increased from 0.158 Nm/kg (0.008–0.432, IQR = 0.128) to 0.471 Nm/kg (0.182–0.960, IQR = 0.191), yielding a median improvement of +0.267 Nm/kg (*p* = 0.001). These findings suggest that Walkbot-assisted training was associated with enhanced torque generation during the stance phase, reflecting improved load-bearing and weight-acceptance mechanics.

### 3.3. Functional Balance

Significant improvements were observed in functional balance following Walkbot-assisted gait training (Table 3). Median Berg Balance Scale (BBS) scores increased from 22.0 (14.0–38.0, IQR = 13) at baseline to 34.0 (25–52, IQR = 15) post-training. This corresponded to a median improvement of +12.0 points (IQR = 5), which was statistically significant (*p* = 0.001). These findings indicate a substantial enhancement in balance performance following the intervention.

### 3.4. Lower Extremity Motor Function

Lower-extremity motor impairment improved significantly following Walkbot-assisted gait training (Table 3). FMA-LE scores increased from 14.0 (8.0–19.0, IQR = 7) at baseline to 24.0 (18–31, IQR = 6) post-training. The median improvement was +10 points (IQR = 4), which was statistically significant (*p* = 0.001). This change indicates a clinically meaningful improvement in paretic lower-limb motor function.

## 4. Discussion

The present study investigated the phase-specific biomechanical and functional effects of robot-assisted gait training using the Walkbot system in individuals with hemiplegia. By focusing on joint torque generation during the swing and stance phases of the gait cycle, this study provides mechanistic insight into how robotic gait rehabilitation may influence lower-limb motor performance and functional balance. The principal findings demonstrate significant torque gains in the affected limb during both the swing and stance phases, accompanied by a substantial improvement in functional balance, as assessed by the Berg Balance Scale (BBS). These findings extend the current evidence base on robot-assisted gait training (RAGT) by linking phase-specific kinetic adaptations to clinically meaningful balance outcomes [24,25,26,27]. Phase-Specific Torque Adaptations and Their Functional Relevance Gait impairments after stroke are characterized not only by altered kinematics but also by profound abnormalities in joint kinetics, including reduced torque generation and inefficient force transmission across the gait cycle [28,29]. While many robotic gait studies focus on spatiotemporal or kinematic outcomes, fewer investigations have examined kinetic variables such as joint torque, despite their central role in propulsion, stability, and energy efficiency [30]. In this context, the present study’s phase-specific torque analysis addresses a recognized gap in the literature and contributes to a more comprehensive understanding of robotic rehabilitation mechanisms. The observed increase in swing-phase torque in the affected limb suggests an improved capacity for limb advancement, foot clearance, and step initiation—key determinants of effective and safe ambulation. Swing-phase deficits are common after stroke and frequently lead to compensatory strategies such as circumduction or hip hiking, which increase metabolic cost and fall risk [31,32]. The present findings support the notion that repetitive, robot-guided stepping with phase-consistent assistance may enhance neuromuscular activation patterns underlying swing-phase control. This interpretation is consistent with neurorehabilitation principles emphasizing task-specific, high-repetition practice as a driver of motor recovery and cortical reorganization [33,34]. Importantly, this study also demonstrated significant stance-phase torque gains in the affected limb, indicating improved weight acceptance and load-bearing capacity. Stance-phase control is fundamental for postural stability and single-limb support, and impairments in this phase are strongly associated with balance deficits and reduced walking independence after stroke [35,36]. Meta-analyses and systematic reviews of exoskeleton-based and electromechanical gait training have reported improvements in balance and mobility outcomes, particularly when robotic interventions are integrated with conventional therapy [25,26,27,37]. The present stance-phase torque findings provide a plausible biomechanical substrate for these functional improvements, suggesting that enhanced force generation during stance may directly contribute to improved postural control and balance.

### 4.1. Biomechanical Adaptations and Balance Recovery

Strength Recovery Versus Coordination and Symmetry Despite robust limb-specific torque gains, post-stroke gait recovery often exhibits a dissociation between improvements in force-generating capacity and the normalization of interlimb coordination or symmetry. Contemporary models of motor recovery distinguish between restitution of motor output (e.g., strength and torque generation) and reorganization of motor control (e.g., timing, symmetry, and adaptability) [38,39]. The present findings align with this framework, suggesting that robot-assisted gait training may preferentially accelerate strength-related adaptations, whereas higher-order coordination processes may require additional time or targeted interventions. This interpretation is supported by systematic reviews indicating heterogeneous effects of RAGT on gait symmetry and coordination, with outcomes influenced by device characteristics, assistance strategies, and training intensity [24,26,40]. Furthermore, biomechanical studies using instrumented robotic orthoses have demonstrated that while robotic guidance can normalize kinematic patterns during training, abnormal joint torque patterns may persist, highlighting the importance of assessing kinetic outcomes to fully understand motor recovery [30,41]. By incorporating phase-specific torque measures, the present study contributes to a growing recognition that kinetic analyses are essential for interpreting the mechanisms and limitations of robotic gait rehabilitation.

One of the most clinically salient findings of this study is the substantial improvement in functional balance, as reflected by a median increase of 27 points on the BBS. This magnitude of change exceeds reported minimal clinically important differences and suggests a meaningful transition toward greater functional independence [42]. Recent meta-analyses have increasingly included balance outcomes and report that robotic gait interventions can yield moderate improvements in balance measures, including the BBS, particularly in individuals with greater baseline impairments [26,37,43]. The link between torque adaptations and balance recovery is likely multifactorial. Enhanced stance-phase torque in the affected limb may improve weight acceptance and stability during single-limb support, while increased swing-phase torque may facilitate smoother transitions between gait phases and reduce destabilizing compensatory movements [13,35]. In addition, the structured, repetitive nature of robotic gait training may enhance sensory integration, anticipatory postural adjustments, and confidence during locomotion—factors that are central to balance recovery after stroke [44,45]. These mechanisms collectively provide a coherent explanation for the observed parallel improvements in biomechanics and functional balance.

### 4.2. Clinical Implications for Robot-Assisted Neurorehabilitation

From a clinical perspective, the present findings have important implications for the design and implementation of robot-assisted gait rehabilitation programs. The demonstration of phase-specific torque gains in both the swing and stance phases supports the use of robotic systems not only as assistive devices but also as tools for targeted motor retraining. Recent evidence syntheses emphasize that the effectiveness of RAGT depends on appropriate patient selection, the timing of intervention, and integration with conventional therapies [24,25,26,40]. Within this context, phase-specific biomechanical assessment may help clinicians identify which aspects of gait are most responsive to robotic training and tailor interventions accordingly. Moreover, the apparent dissociation between strength recovery and coordination suggests that robotic gait training may be most effective when combined with complementary approaches targeting symmetry, adaptability, and overground transfer. Such multimodal strategies are increasingly recommended in contemporary neurorehabilitation frameworks and may maximize functional carryover to daily walking and community ambulation [46,47]. Importantly, phase-specific torque gains were accompanied by significant improvements in FMA-LE scores, suggesting that biomechanical adaptations may reflect underlying motor recovery rather than solely compensatory mechanisms.

### 4.3. Motor Recovery and Biomechanical Adaptations

An important finding of the present study is that the observed phase-specific torque gains were accompanied by significant improvements in lower-extremity motor impairment, as reflected by the Fugl–Meyer Assessment—Lower Extremity (FMA-LE). Participants demonstrated a median improvement of +10 points, indicating a clinically meaningful enhancement in paretic-limb motor function. Improvements of a similar magnitude have been associated with functional motor recovery and improved voluntary control in post-stroke populations [24,48].

The concurrent improvement in FMA-LE scores suggests that the increases in torque generation were not merely mechanical or device-driven effects but may represent true neuromuscular recovery. The FMA-LE is widely regarded as a stroke-specific measure sensitive to changes in voluntary motor control, synergy reduction, and selective movement capacity [49]. Therefore, the parallel enhancement of kinetic outputs and FMA-LE scores supports the interpretation that Walkbot-assisted gait training facilitated restorative motor adaptations rather than solely compensatory strategies. This interpretation is consistent with recent systematic reviews indicating that robot-assisted gait training (RAGT) can induce both functional and impairment-level improvements when sufficient training intensity is provided [24,43].

### 4.4. Strength Recovery Versus Motor Control

Post-stroke gait improvements often reflect a complex interplay between strength recovery and motor control reorganization. While increased torque generation may arise from improved muscle force capacity, meaningful functional recovery requires refinement of inter-joint coordination, timing, and selective motor activation. In the present study, significant gains in FMA-LE scores indicate improvements in motor control at the impairment level, strengthening the mechanistic interpretation of torque changes.

Importantly, this finding reduces the likelihood that the torque increases observed in both the swing and stance phases were driven exclusively by contralateral compensation. Instead, improvements in paretic-limb motor performance suggest enhanced voluntary recruitment and modulation of lower-limb musculature during gait. Contemporary neurorehabilitation frameworks emphasize that recovery characterized by improved selective motor control reflects restitution rather than compensation [43].

### 4.5. Neuroplastic Mechanisms

The association between torque gains and FMA-LE improvement may be explained by neuroplastic processes induced by repetitive, task-specific robotic gait training. High-repetition stepping with phase-consistent assistance has been shown to promote cortical reorganization, strengthen sensorimotor integration, and enhance residual descending motor pathways [43].

Robotic systems such as Walkbot provide consistent proprioceptive feedback and error-reduced practice, both recognized drivers of experience-dependent neural plasticity. Recent meta-analyses and neurophysiological investigations further suggest that robotic gait interventions may modulate motor cortex excitability and improve motor network efficiency following stroke [43].

The improvement in FMA-LE scores observed in this cohort aligns with this framework, suggesting that robot-assisted gait training may contribute to recovery at both the biomechanical and neural levels.

### 4.6. Limitations

Several limitations should be acknowledged. First, the small sample size, particularly in the left hemiplegic group (*n* = 5), may limit generalizability and statistical power for between-subgroup comparisons. Second, because subacute stroke patients may demonstrate spontaneous motor recovery, the observed biomechanical and clinical improvements cannot be attributed entirely to Walkbot-assisted gait training. The absence of a non-intervention control group limits the ability to dissociate intervention effects from natural recovery trajectories. Third, our study did not assess long-term retention of improvements or functional transfer to community ambulation.

### 4.7. Methodological Considerations and Future Directions

The pre–post design without a control group limits causal inference, and the modest sample size reflects the exploratory and mechanistic nature of this study. Nevertheless, the consistent phase-specific torque gains and robust balance improvements provide compelling preliminary evidence of training-related adaptations. Importantly, recent meta-analyses highlight a persistent need for studies that report biomechanical outcomes alongside clinical measures to improve mechanistic understanding and inform optimization of robotic protocols [24,26,37]. Future research should incorporate controlled designs, larger samples, and longer follow-up periods to evaluate retention and generalization of torque gains. Additionally, integrating kinetic analyses with neurophysiological and kinematic measures may further elucidate how robotic gait training influences motor recovery at multiple levels of the neuromuscular system.

## 5. Conclusions

In summary, Walkbot-assisted gait training was associated with improvements in phase-specific torque, functional balance, and lower-limb motor recovery in individuals with hemiplegia. These results highlight the relevance of phase-resolved kinetic assessment for capturing biomechanical adaptations during robotic rehabilitation. However, given the pilot design and inclusion of subacute participants, causal interpretations should be made cautiously. Future randomized controlled trials are needed to validate and extend these findings.

## Figures and Tables

**Table 1:** System domains, sensing and control components, measured parameters, and relevance to gait analysis and rehabilitation.

Domain	Component/Feature	Sensor Type/Source	Measured Parameters	Relevance to Gait Analysis and Rehabilitation
Joint actuation	Hip, knee, ankle modules (bilateral)	Electric motors with integrated encoders	Joint position, angular velocity	Enables precise joint-level control and continuous kinematic monitoring during gait
Torque estimation	Joint torque sensing	Embedded torque sensors and motor current-based estimation	Joint torque (Nm), phase-specific torque profiles	Provides objective kinetic biomarkers of force generation during swing and stance
Gait-phase detection	Phase recognition module	Sensor fusion (joint kinematics + temporal thresholds)	Swing- and stance-phase identification	Allows phase-specific assistance and segmentation of biomechanical data
Kinematic sensing	Joint angle measurement	High-resolution rotary encoders	Joint angles (°), angular velocity (°/s)	Quantifies movement quality and coordination across gait phases
Spatiotemporal analysis	Gait-timing computation	Encoder-derived temporal signals	Step timing, cadence, phase duration	Supports assessment of temporal gait symmetry and rhythm
Control strategy	Assistive control	Position- and impedance-based control algorithms	Assistance level, joint impedance	Enables adaptive, patient-specific robotic assistance
Training dose monitoring	Session logging	Internal system logger	Training duration, speed, body-weight support	Facilitates dose–response and longitudinal analyses
Data acquisition	Sensor synchronization	Central data acquisition unit	Time-synchronized kinematic and kinetic signals	Ensures reliable offline biomechanical analysis
Clinical integration	Outcome linkage	Software-based data export	Torque–balance associations (e.g., BBS)	Links sensor-derived metrics with functional outcomes

**Table 2:** Demographic and etiological characteristics of the patients.

Variable	Total (*n* = 15)
Age, years (mean ± SD)	58 ± 7
Sex, *n* (%)	
Female	6 (40%)
Male	9 (60%)
Stroke type, *n* (%)	
Ischemic	5 (33.3%)
Hemorrhagic	10 (66.7%)

**Table 3:** Pre- and post-intervention clinical and kinetic outcomes.

Outcome Family	Variable	Pre Median (Min–Max, IQR)	Post Median (Min–Max, IQR)	*p*	Δ Median (IQR)
**Clinical**	BBS score (all)	22.0 (14.0–38.0, 13)	34.0 (25–52, 15)	0.001	12.0 (5)
	FMA-LE score	14.0 (8.0–19.0, 7)	24 (18–31, 6)	0.001	10 (4)
**Kinetics (Swing)**	Affected torque (Nm/kg)	0.261 (0.084–0.458, 0.161)	0.361 (0.117–0.767, 0.216)	0.017	0.095 (0.148)
	Unaffected torque (Nm/kg)	0.254 (0.058–0.440, 0.131)	0.334 (0.202–0.740, 0.182)	0.011	0.136 (0.195)
**Kinetics (Stance)**	Affected torque (Nm/kg)	0.197 (0.005–0.512, 0.232)	0.454 (0.208–0.851, 0.350)	0.001	0.197 (0.237)
	Unaffected torque (Nm/kg)	0.158 (0.008–0.432, 0.128)	0.471 (0.182–0.960, 0.191)	0.001	0.267 (0.271)

## Data Availability

The data presented in this study are available on request from the corresponding author. (The data are not publicly available due to privacy or ethical restrictions).
